# Author Correction: microRNA165 and 166 modulate response of the Arabidopsis root apical meristem to salt stress

**DOI:** 10.1038/s42003-023-05245-8

**Published:** 2023-08-29

**Authors:** Daria Scintu, Emanuele Scacchi, Francesca Cazzaniga, Federico Vinciarelli, Mirko De Vivo, Margaryta Shtin, Noemi Svolacchia, Gaia Bertolotti, Simon Josef Unterholzner, Marta Del Bianco, Marja Timmermans, Riccardo Di Mambro, Paola Vittorioso, Sabrina Sabatini, Paolo Costantino, Raffaele Dello Ioio

**Affiliations:** 1 Dipartimento di Biologia e Biotecnologie Charles Darwin, Università di Roma, Sapienza - via dei Sardi, 70, 00185 Rome, Italy; 2 Department of Biology, University of Pisa, via L. Ghini, 13, 56126 Pisa, Italy; 3 Center for Plant Molecular Biology, University of Tübingen, Auf der Morgenstelle 32, Tübingen, 72076 Germany; 4 Faculty of Science and Technology, Free University of Bozen-Bolzano, Piazzale Università, 5, 39100 Bolzano, Italy; 5 Italian Space Agency, Rome, Italy

**Keywords:** Salt, Root apical meristem, Abiotic

Correction to: *Communications Biology* 10.1038/s42003-023-05201-6, published online 11 August 2023.

In this article the author name Simon Josef Unterholzner was incorrectly written as Simon Joseph Unterholzener. The original article has been corrected.

