# Supplementary material for: microRNA165 and 166 modulate response of the Arabidopsis root apical meristem to salt stress
Source: Commun Biol. 2023 Aug 11;6:834. doi: 10.1038/s42003-023-05201-6 (PMC10421904; doi:10.1038/s42003-023-05201-6)
Supplement: Supplementary file 2 — Supplementary Materials [file 42003_2023_5201_MOESM2_ESM.pdf]

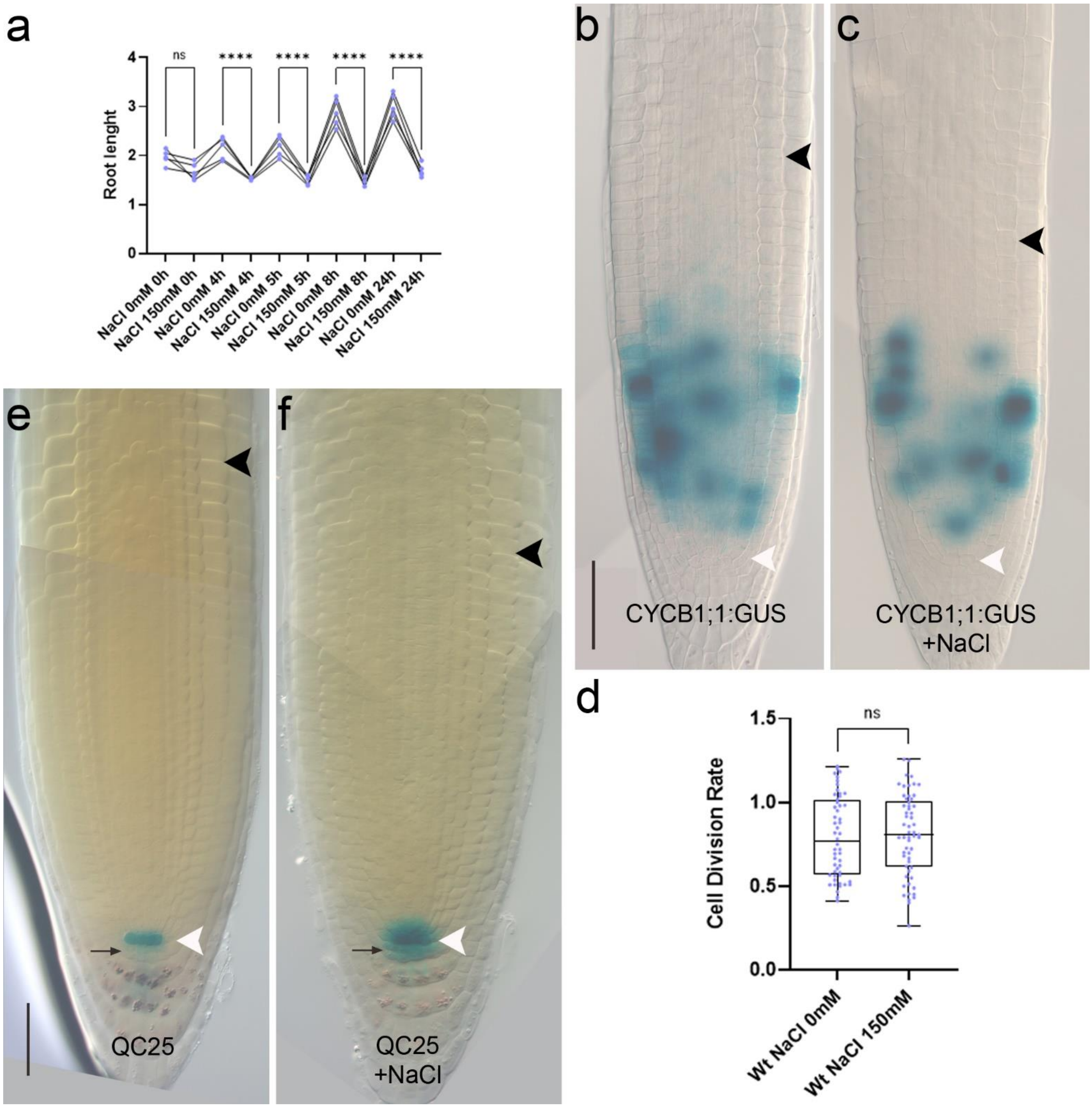

Supplementary Figure 1: Salt stress inhibits root growth and meristem function regulating cell differentiation activity.

a) Root length over time of Wt plants grown on standard MS medium in comparison to Wt plants exposed to 150 mM NaCl . (ns not significant; ****p<0.001; One-way ANOVA with post hoc Sidak multiple comparisons tests; n =5)

b-c) DIC images of the histochemical b-glucuronidase (GUS) assay detecting CYCB1;1:GUS localization at 5dpg in a Wt root meristem upon mock and NaCl (NaCl 150mM for 5 h) treatments. Scale Bar 100 µm, white arrowheads indicate the cortical stem cell, black arrowheads the TZ.

d) Graph showing no reduction in cell division rate (GUS stained cells/meristem cell number) after NaCl treatment (NaCl 150mM) in Wt plants (Col-0 background; ns not significant; Student's t test; n=52, 57). Box and whiskers plots show the median, 25th and 75th percentile (box limits), the 10th and 90th percentiles (whiskers), and outliers points.

e-f) DIC images of the histochemical GUS assay performed in *QC25::GUS* plants upon mock and NaCl (NaCl 150mM for 5 h) treatments. Scale Bar 100 µm; white arrowheads indicate the cortical stem cell, black

arrowhead the TZ. Black arrows indicate columella stem cells.

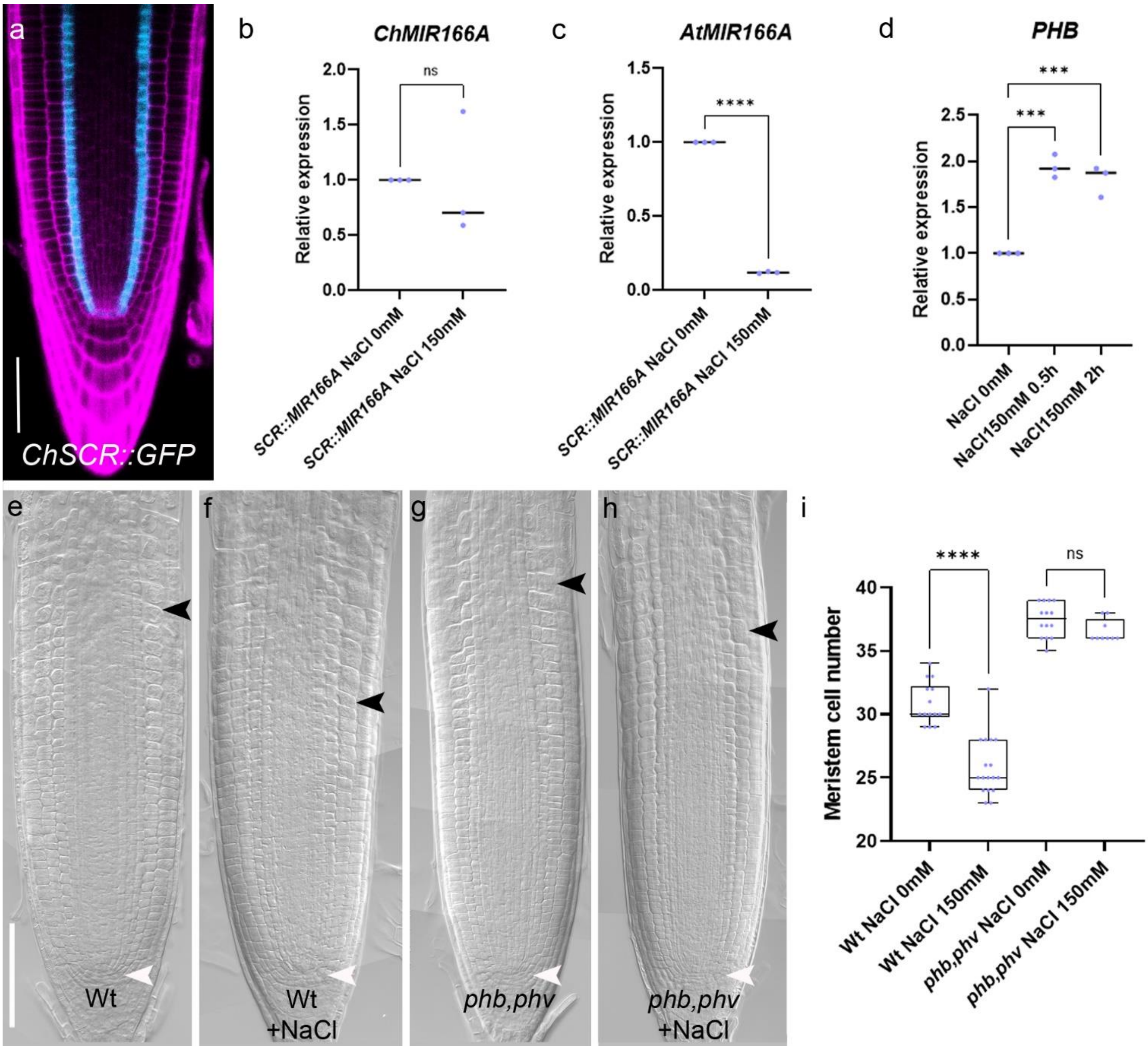

Supplementary Figure 2: miR165 and 166 regulate root meristem size in response to salt stress.

a) Expression of *ChSCR::GFP* in 5-DAG root meristem of Wt plants (Col-0 background). Scale Bar 50 µm.

b-c) qRT–PCR analysis of *ChpreMIR166A* (b) and *AthpreMIR166A* (c) RNA levels in the root tip of *ChSCR::MIR166A* plants upon 150 mM NaCl for 2 h (ns not significant ; ****p<0.0001; Student's t test;n=3).

d) qRT–PCR analysis of PHB RNA levels in the root tip of Wt plants upon 150 mM NaCl for 30 minutes and 2 h (***p < 0.001, **** p<0.0001; Student's t-test; n=3)

e-h) DIC images of 5 dpg root meristems of Wt (d), Wt exposed to 150 mM NaCl for 5 h (e), *phb,phv* (f) and *phb,phv* plants exposed to 150 mM NaCl for 5 h (g). Scale Bar 100 µm; white arrowheads indicate the cortical stem cell, black arrowheads the TZ.

i) Root meristem cell number of Wt and *phb,phv* root meristems exposed to 150 mM NaCl for 5 h (ns not significant, ****p<0.00001, One-way ANOVA with post hoc Sidak multiple comparisons tests; n=14, 17, 14, 9). Box and whiskers plots show the median, 25th and 75th percentile (box limits), the 10th and 90th percentiles

(whiskers), and outliers points.

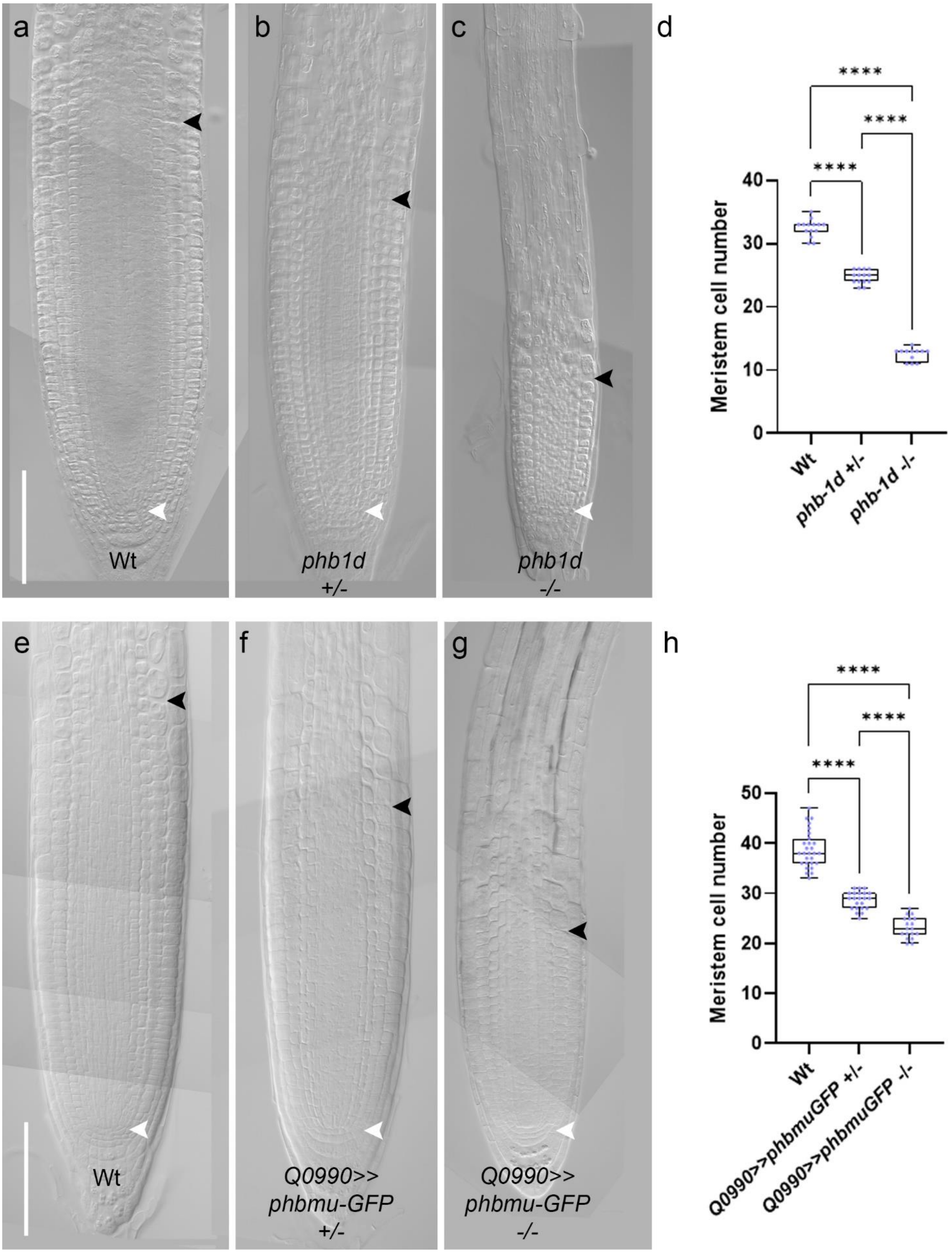

Supplementary Figure 3: PHB controls root meristem size in a dose dependent manner.

a-c) DIC images of 5 dpg root meristems of Wt (a), *phb1d* heterozygous (d) and *phb1d* homozygous plants. Scale Bar 100 µm, white arrowheads indicate the cortical stem cell, black arrowheads the TZ (n=14, 14, 12).

d) Root meristem cell number of Wt, *phb1d* heterozygous and *phb1d* homozygous root meristems at 5 dpg

(****p<0.0001; One-way ANOVA test with Tukey's multiple comparisons test; n=14, 14, 12). Box and whiskers plots show the median, 25th and 75th percentile (box limits), the 10th and 90th percentiles (whiskers), and outliers points.

e-g) DIC images of 5 dpg root meristems of Wt (e), *Q0990>>phbmu-GFP* heterozygous (f) and *Q0990>>phbmu-GFP* homozygous plants (g). Scale Bar 100 µm, white arrowheads indicate the cortical stem cell, black arrowheads the TZ (n=28, 22, 18).

h) Root meristem cell number of Wt, *Q0990>>phbmu-GFP* heterozygous and *Q0990>>phbmu-GFP* homozygous root meristems at 5 dpg (****p<0.0001; One-way ANOVA test with Tukey's multiple comparisons test;n=28, 22, 18). Box and whiskers plots show the median, 25th and 75th percentile (box limits), the 10th and 90th percentiles (whiskers), and outliers points.

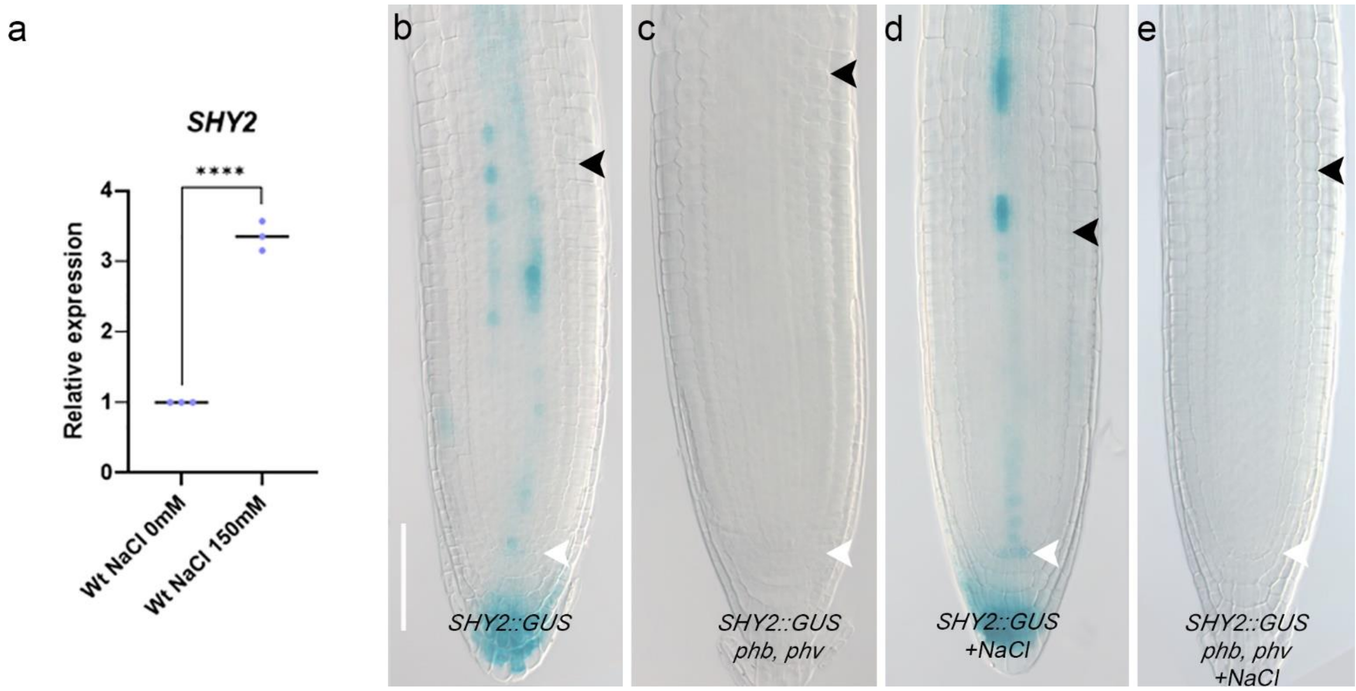

Supplementary Figure 4. Salt stress promotes the expression of *SHY2* in a *PHB* and *PHV* dependent manner.

a) qRT–PCR analysis of *SHY2* RNA levels in the root tip of Wt plants upon 150 mM NaCl for 2 h (****p<0.0001; Student's t test; n=3).

b-e) DIC images of the histochemical b-glucuronidase (GUS) assay detecting *SHY2::GUS* expression at 5dpg in a Wt root meristem upon mock (d) and NaCl (NaCl 150mM for 5 h; (f) treatments. Scale Bar 100 µm, white arrowheads indicate the cortical stem cell, black arrowheads the TZ (n=20).
